# Hospital and Institutional News

**Published:** 1915-02-13

**Authors:** 


### HOSPITAL AND INSTITUTIONAL NEWS.

### THE POOR-LAW CONFERENCE.

On Tuesday last the forty-third Annual Conference of Poor-Law Guardians of England and Wales was opened in the Council Chamber of the Guildhall. The delegates, who were welcomed by the Lord Mayor and addressed by Mr. Herbert Samuel, numbered 250. After the address of the President of the Local Government Board, which is dealt with in our second leading article, two papers on "The Relief of Distress during War" were read, and Tuesday afternoon was devoted to their discussion. On Wednesday, after a paper on "The Local Government Board Circular on Relief to Widows and Children," the afternoon was given up to a discussion on "The Poor Law in Relation to the Blind and other Physical Defectives," by the Rev. G. A. Suttle, the chairman of the West Ham Board of Guardians. We hope next week to deal with Mr. Suttle's remarks and the discussion which ensued upon them, seeing that they form the feature of the Conference with which institutional officers are most likely to be concerned. So many aspects of the Poor Law present themselves for treatment at these Conferences that questions arising out of institutional administration and treatment are usually represented directly only in one paper or discussion.

### AN APPEAL FOR VOLUNTARY RESIDENTS.

How serious is the shortage of resident medical officers for some institutions may be gathered by the appeal which appeared in the columns of the *Lancet* last week, when Mr. W. M. Wilcox, secretary of the East London Hospital for Sick Children, Shadwell, actually stated that, unless help in this direction were obtained, a large part of the hospital would have to be closed. Here are his actual words: "It is possible there may be doctors who, if they knew of the difficulties under which hospitals are now labouring, would be willing to come into voluntary residence and help them during this temporary crisis. We have exhausted all the regular methods of obtaining resident doctors, and unless we can procure such help we shall be compelled to close a large part of the establishment, which at the present time would be a great calamity for the sick children." This is, it appears, a novel attempt at solving the existing difficulty, and it will be interesting to see with what success it meets, and if the example which Mr. Wilcox has set is to be followed. It is at all events at the opposite extreme of doubling previous salaries, but how far the profession, even at a crisis, would see its way to adopt this new departure remains to be seen.

### HOW TO RAISE MONEY FROM ABANDONED ANNUAL EVENTS.

Any sidelight on this subject is helpful at the present time, although we have already given reasons for thinking that hospitals have really as good an opportunity now as before the war for raising money, if they go to work in the right way. Thus in connection with the East Sussex Hospital a ball was held last year, and deemed sufficiently successful to become an annual event. Owing to the war it is being abandoned, but that the hospital may not lose, all those who took tickets last year have been asked to send a sum equivalent to the value of those bought twelve months ago. The first half of the total profit was quickly raised, and by the time that these lines are in print, no doubt the former total will have been realised. Of course, the plan applies to many other hospital events besides balls; and its only defect appears to be that no new givers are appealed to. But, on the other hand, the charitable public is a small one. Those who give once, give again; and the real aim of charitable finance is how to extract money from those who have hitherto not responded to appeals. At all events, as a suggestion for getting over the difficulty of abandoned annual events caused by the war, we quote the success of the above for what it may be worth.

### AN EMERGENCY SURGICAL SERVICE.

A new voluntary branch of medical service has sprung into being out of the possibility of further air raids on our shores. To meet the risks arising therefrom to the civil population a committee of Fellows and Members of the Royal Society of Medicine has organised, with the approval of the War Office and the Commissioner of Police, a voluntary service of surgeons, assistants, and anæsthetists. On being called out these are prepared to start immediately to any point on the coast from the Wash to Dover, or to any part in the Metropolitan area. By the assistance of the Royal Automobile Club a fleet of cars has been placed at the committee's disposal, which can convey the complete parties, with their individual supply of surgical appliances, free to any London district; or, in case the Great Eastern Railway, which has offered special trains where possible, might be blocked for military purposes, to the East Coast direct. This is the response of the medical profession to the threatened air raid, and it should be of value in supplementing the possible disorganisation which might prevail at the centre where such a raid may occur.

### ARE THE VOLUNTARY AID DETACHMENTS ABUSED?

In the days when the story of the arrival of large batches of wounded at the different stations was new, and interest was centred on the quality of the preparations made by the local Voluntary Aid Detachments, it was often remarked how promptly these responded to a sudden call to prepare for the arrival of a large convoy, and also, not infrequently, how these convoys did not arrive until the next day or sometimes even later. These delays were taken philosophically, the Voluntary Aid members consoling themselves by the value of the impromptu "drill" they had undergone; and no one ventured to criticise the military authorities.

To-day, when the arrival of convoys is almost a part of the week's work, and has lost its novelty, this spirit of sufferance is beginning to break down, and people are beginning to ask themselves whether it really is so impossible for the military authorities to know whether or not a convoy is arriving at a certain place at a certain time. The private motor-car owners, who lend their cars on these occasions, have begun to protest, as the following published statement shows: "If there is a feeling that the owners of cars generally in the district are not doing all they might, the military authorities have only themselves to blame. . . . On one occasion I found out that the train would be an hour late, and was proceeding to make arrangements to telephone to the drivers of the ambulances that the train would be late, when a transport officer informed me it was quite unnecessary to telephone to them, as it would do them no harm to wait at the station. Furthermore, the military authorities very often ask for more cars and ambulances than are absolutely necessary, which has discouraged to my certain knowledge a great many of the drivers, as no man likes to turn out at 3 A.M. to find he is not wanted." There probably has been a good deal of unnecessary carelessness in this matter, and it is only right that the voluntary workers should ask for reasonable recognition of their convenience.

### THE ANOMALOUS POSITION OF THE WORKHOUSE MEDICAL OFFICER.

When the Poor-Law Institutions (Draft) Order was under discussion, and again when the Order itself was promulgated, we drew attention to the anomalous position occupied by the workhouse medical officer. We pointed out that whilst he was necessarily made responsible for the treatment of the sick inmates the clauses of the Consolidated Order making him subordinate to the workhouse master had been re-enacted in a most stringent form. The medical officer was thus left without any power to insist upon the carrying out of many orders he might give in the interests of his patients. We predicted at the time that unless great tact were exercised by both medical officer and workhouse master conflicts would arise in which the patients would suffer. A recent occurrence at the Epping Workhouse confirms our prediction. Divested of the personal recriminations which are always bound to arise under such circumstances the facts, which are not disputed, are as follows: A patient in the workhouse developed symptoms necessitating an immediate operation. This was carried out, and at the end of the operation the medical officer telephoned for men to be sent at once to remove the patient to the ward. A reply was sent back that all the officers and men were in the dining-room and could not be sent at once. The medical officer again telephoned that the patient must be removed at once, and received the reply from the master that the matter was being attended to. Finally, after about twelve minutes' delay, when the meal in the dining-room was over, the men were sent. The medical officer then sought out the master's clerk, to whom the messages had been sent, and reprimanded him sharply for the delay. The master objected to this, pointing out that as he was the chief officer the medical officer had no right to reprimand any of the officers.

### THE MORAL OF THE EPPING DISPUTE.

Technically the master was undoubtedly in the right, and he was supported by the Board of Guardians, who passed a vote of censure upon the medical officer for ignoring the master's authority. The position is obviously an utterly impossible one. The medical officer is left without any shred of authority. His orders may be ignored or carried out perfunctorily or after unnecessary delay, as in this case, and his only redress is a complaint to the house committee, which may not meet for a week or two afterwards. Even if the committee supports him, it is the patients who suffer in the meantime. Responsibility divorced from authority leads to general defective administration. It causes loss of discipline and slackness in the performance of duties. It affects the whole tone of the establishment, to the detriment of the inmates. There can be no satisfactory solution until the workhouse medical officer is made the administrative head of the sick wards.

### THE VISITATION OF PATIENTS IN MENTAL HOSPITALS.

The Lambeth Board of Guardians for many years have not visited any of the mental hospitals to which patients from the borough are sent. They felt that as the institutions were under the control of public authorities, who appointed responsible committees to administer and look after them, there was no necessity for the Guardians to send deputations to visit the patients, as it might hamper the work of the staffs. The Guardians have now received a letter from the Board of Control respecting the non-visitation by the Guardians of their patients in Long Grove, in which they express strongly their views that the visitation by Guardians of patients from their Unions is of the first importance to the comfort and contentment of the patients who, cut off and separated from their homes and friends, and suffering frequently from a sense of injustice at their confinement at all, regard with much pleasure the visits of the outside local authorities, who bring them into some sort of relation again with their own homes. The letter adds that this has been expressed on very many occasions to the Commissioners by patients, and they knew it to be a wide-spread feeling which was deserving of practical recognition. The visits of the Mental Hospitals Committee, useful as they were, did not at all replace those of the Guardians, and could not be regarded as a substitute for them, and the Board earnestly hoped, therefore, that the Lambeth Guardians would make a point of securing the regular visitation of the mental patients chargeable to their Union who were confined to institutions.

### THE VALUE OF GUARDIANS' VISITS.

In view of the foregoing representations of the Board of Control, the Guardians decided again to resume their old practice of periodically visiting the mental hospitals. In many reports presented to the Guardians by the deputations visiting the mental hospitals, the complaint often occurs that the patients have not only not been visited by anyone from their own parish since the last time a deputation attended, but have not seen any of their relatives; indeed, have not heard from them for twelve months. It has been found that by the visits relatives have been awakened to a due sense of their responsibilities to those in the mental hospitals. Again, by judicious questioning and an investigation afterwards into the replies, it has been found there were close relations to the patients who were well able to contribute something towards the patients' maintenance in the institution. At all events, the visitations by committees from the Boards of Guardians remind them that they are not altogether forgotten by those from the parish in which they were born and spent many years of their lives. In agreeing to step into line with the other Metropolitan Boards of Guardians, Lambeth has taken a step which will bring comfort to many of the 519 patients from the parish now residing in the institutions of the London County Council.

### THE NEW OUT-PATIENT DEPARTMENT AT THE ROYAL FREE HOSPITAL.

On Monday the new out-patient department at the Royal Free Hospital was opened. We will not repeat descriptions which have already appeared in our columns of the new unit, which is composite in the sense that a variety of departments has been grouped in it, although the extension is not yet complete. A massage department and new wards have yet to be added, but the opening marks an important stage in the large extension scheme which was decided on some time ago, and of which the previous stages have been noted already.

### HOSPITAL SECRETARY'S FINE RECORD.

The general court of governors of the Royal Infirmary, Sunderland, held last Monday, had particular interest from the fact that the committee record the long period of service rendered by the secretary, Mr. Thomas Robinson. For thirty-two years Mr. Robinson has held this post, and the committee remark that his "zeal and devotion throughout this prolonged period of many and varied changes has been a service of strength to the infirmary and of complete satisfaction" to the managers. It is indeed a long and fine record as the following facts succinctly show. In 1882 in-patients numbered 1,169; they amount to 4,888 to-day. The total income has risen from five to seventeen thousand pounds, now as then being slightly in excess of the expenditure. And when we turn to the capital account we find that buildings and investments combined, while thirty-two years ago they amounted to £36,000, to-day—that is, in 1914—have reached the great sum of £128,000. Hospital readers will fill in the body, of which these facts are but the skeleton, and congratulate Mr. Robinson on his long record, the younger men perhaps envying him the experience stored up by the exercise of responsibility during so many changes and from so much growth.

### THE SANATORIUM LETTER-WRITER.

It has been necessary from time to time to record the complaints made by sanatorium patients against their treatment, diet, or discipline, particularly in the case of those received in institutions through the operations of the National Insurance Act. We have even published correspondence dealing with the administrative aspects of such complaints; and the idea has become prevalent that the insured tuberculous person belongs to a disgruntled class. It is, therefore, only fair to record a sample of their gratitude, which, our readers will note, is as profuse as the complaints often quoted are severe. The sanatorium concerned is the institution at Bromley, lent during the end of 1914 by the Bromley and Beckenham Joint Hospital Board for the treatment of insured consumptives, who have since been removed to Benenden so that the Bromley institution may be prepared for the treatment of soldiers suffering from enteric. Twenty one of the sanatorium patients drafted a letter addressed to the clerk to the Joint Hospital Board, in which the following extract occurred: "We can assure you that in so far as happiness is compatible with the gloom that overhangs our country, and with our own somewhat unfortunate circumstances we indeed had a most happy Christmas. The means of entertainment and the good cheer that you have so generously provided have afforded unbounded enjoyment. We also desire to offer our most heartfelt gratitude to the whole of the sanatorium staff." It is to be noted here that the diet is singled out for approval, and the whole affords a pleasing example of the sanatorium letter-writer in his happiest vein; and letter-writing is a pursuit often encouraged by sanatorium treatment in those already possessed of an inclination in that direction.

### THE IMPATIENT RECRUIT.

He was a would-be recruit for His Majesty's Army, but had been rejected by the medical officer for slight physical disability, which it was intimated an operation would remove. He was accordingly recommended to the local hospital for the special ward dealing with recruits. On the morning arranged for admission came the following postcard, literally transcribed: "Sir,—My husband as decided not to have an opperation, if they cannot take him to fight for his King and country without an opperation they must do without my husband.—Mrs. Arthur."

### THE DUAL APPOINTMENT OF MARRIED COUPLES.

The Jarrow Town Council have recently considered the terms of appointment of a porter and matron at their infectious hospital. Hitherto it has been the rule at Jarrow that these appointments should be held by a married couple, and an attempt was made to enforce the same rule in the future. The majority of the Council, however, decided that it would be better to word the advertisement so as to leave the posts open both to married couples and single applicants. We think the Council decided wisely, and that it would have been even better to have reversed the old rule and to have decided that the two posts should not be held by a married couple. The qualifications for them are so widely different that to make them a dual appointment has the effect of restricting the choice to a very limited circle. We go further and say that a dual appointment of this kind is entirely out of date. The position of the matron of a modern hospital is such that she ought not to be the wife of its porter. The work required of her is such that she cannot carry them out satisfactorily and at the same time perform her duties as a wife and mother. It is curious that the Local Government Board still hold to this rule in the case of the master and matron of a workhouse. Recently the St. Pancras Board of Guardians have had the greatest difficulty in inducing the Board to sanction the appointment to these posts in the case of the local workhouse of two excellent and experienced officials who were not married to one another. Fortunately the Guardians have at last induced Mr. Samuel to recognise the reasonableness of their request, and have drawn from him the declaration that he will have a departmental inquiry made into the general question. Dual appointments no doubt originated from motives of economy. The arguments against them—that they bar out many candidates eminently fitted for one or other of the posts, that in many cases they interfere with normal married life to the detriment of the individual and of the State, that the discipline of the institution suffers from them, and that the death of one may entail unnecessary hardship upon the other—are unanswerable. Reflections of this kind have led many managing bodies to abolish such posts, and it is to be hoped that the system, except in certain special instances, will disappear entirely.

### THE YORK FEVER HOSPITAL EXTENSION SCHEME.

Several points of interest occur in the exhaustive report which Dr. Edmund H. Smith, medical officer of health, has presented to the Health Committee of York City Council in connection with the proposal to extend the Fever Hospital in Huntingdon Road at a cost of £30,000. The present institution comprises two ward pavilions, administrative block kitchens, an open-air ward, and a house block. Dr. Smith points out that very inadequate accommodation, causing a selection of scarlet fever cases, which wastes much time; the want of two separate blocks for new cases of scarlet fever, and a separate block for these cases when convalescent, the want of private wards, and the worn-out and over-septic condition of the present scarlet fever block are cardinal defects of the existing institution. He points out that extensions were suggested in 1892, and he now urges that one of the new acute scarlet fever blocks should be at once begun at a cost of £4,000; that a new laundry block, with a steam-machine laundry, boiler-house, and drying-ground, should be built; that an entirely new administrative block (including seventeen single bedrooms for nurses and ten bedrooms for maids) should be erected at a cost of £5,500; and that the adaptation of the present scarlet fever block for advanced phthisis, and of the present administrative block as a discharging house, should follow.

### MODERN PLANNING OF FEVER HOSPITALS.

Into the second half of the extension scheme outlined in the report it would be premature to enter, but his plea as an expert to be heard and attended to is one with which his colleagues will sympathise at the present time. "Over and over again during the past years," he writes, "I have had to regret the delay in carrying out the Fever Hospital extension, and that delay might well be subject for criticism; but regret is considerably tempered by the fact that, in the meantime, the open-air treatment and other methods have developed to such an extent as to greatly modify one's ideas of designing a hospital. My prolonged experience, and that of other medical superintendents of hospitals, has also modified one's ideas, so that to-day one really finds that there have been some advantages in the delay, and that a more modern hospital should be the result." There must, however, be a limit to this process, and we hope the York authorities, in the light of this report, will feel that it has now arrived.

### THE NEW PATHOLOGICAL DEPARTMENT AT LEICESTER.

An excellent start has been made with the fund for the erection of a new pathological department and mortuary in connection with the Leicester Royal Infirmary, and it must have been a source of encouragement to the new chairman, Mr. H. Simpson Gee, to announce that Sir Edward Wood had shown his continued interest in the institution by offering to contribute £1,000 to the new building. To this amount Mr. Gee himself added a contribution of £500, and Mr. C. J. Bond, the deputy chairman, one of £100, so that practically one-half of the £3,600 required has been raised. We hope the "merchants" of Leicester, who are profiting by the pressure of Government orders, will quickly respond to the appeal for generous gifts to this necessary new departure for a well-equipped institution.

### MIDDLESEX COUNCIL AND ITS PROPOSED SANATORIUM.

The Middlesex County Council have had under consideration the question of the erection of a sanatorium at a new site, Mayland. The cost is estimated to be approximately £70,000. This is some £12,000 in excess of the estimate for the original site on which it was proposed to erect a sanatorium. Of course, since then building materials and labour have advanced in cost greatly, owing to the war, and the Government would contribute about £30,000 towards the cost of the scheme. Opposition, however, was given to the proposal on the grounds that the Middlesex County Council were induced to pass the first scheme because the work would provide employment for the large number of workmen in need of it owing to the war. At present about 300 Middlesex patients are having institutional treatment, and another 100 are on the waiting list, besides a number in Poor-Law infirmaries. Eventually, after much discussion, the scheme was deferred, except that the loan required for the land was approved. If the Government are satisfied with the scheme proposed—as they would appear to be if they have promised a grant—there seems no valid reason why this work should not have been put in hand, especially in view of the fact that the provision for institutional treatment for tuberculosis is quite inadequate, as shown by the committee's report.

### HIGHER SALARIES AT BIRMINGHAM UNION INFIRMARY.

Some time ago the Guardians of the Birmingham Union revised the hours of duty and leave of absence of their nursing staff. They have now carried the matter a step further and have revised the scale of salaries of charge nurses and probationer nurses, and have also standardised throughout their three infirmaries the periods of probationers' training as follows: The charge nurses' salaries are now £38, increasing by annual increments of £2 to a maximum of £42 per annum. As to probationers, the period of training will be one of three years and three months, including the three months' trial period, which will be paid for, and the salary will be £12 for the first year, £15 for the second year, and £18 for the last fifteen months. A selection will be made from the best probationers, as vacancies occur in the maternity schools for them, to obtain midwifery training and qualify for the C.M.B. Probationers so chosen will remain an extra nine months, making four years in all, and they will receive £18 for the third year only and £26 for the fourth year, with the rank of staff nurse. The salary for the fourth year is increased more than usual to allow the nurse to pay expenses she will incur in taking some of her maternity training outside the institution under an arrangement the Guardians have made with two qualified teachers. The Guardians deem these salaries and training periods to be quite in keeping with the modern trend of thought of the nursing world, and they are certainly a further step in the right direction worth notice by other boards and hospital administrators generally. The movement for improved salaries among institutional staffs has been already too long delayed.

### "THE EVOLUTION OF THE MODERN HOSPITAL": A NEW SERIES.

The interest of hospital workers, not only in the history of their institutions, but also in the broader aspects of the growth of the voluntary principle in the institutional treatment of disease, is one which we have done much to encourage. The hospital history is at last becoming a recognised necessity for each institution to possess, and the series of articles on "The Eighteenth Century Hospital Movement," recently published, evoked so much interest that we feel that our readers will appreciate an intimation that another series of historical articles, this time from the pen of Mr. Burford Rawlings, is shortly to appear. Their subject is "The Evolution of the Modern Hospital," and the first article of the series begins by a striking comparison of present conditions with those which prevailed forty years ago, a subject on which, as a hospital secretary now retired from active work, the writer can throw a vivid light from personal memory. It is well at the present time that the public, which is now glorying in the work which the voluntary hospitals are doing for the wounded, should be reminded how great have been the efforts in which modern improvements have resulted, and how needful is more public support if progress is to be maintained. Such details as the evolution of the type of patients' lockers give point to the story, which, in its larger aspect, includes a discussion of such questions as whether lay or medical authority ought to be supreme in institutions both philanthropic and educational. This involves a consideration of the relation of medical education to hospital administration and finance, which is dealt with not only theoretically, but in the light of events in recent history. Questions of the payment of hospital medical staffs, the desirability of increasing the numbers of the latter, and the value of medical superintendents are also touched on as the central theme of the evolution of the modern hospital provides occasion. Round this story of development these debatable points are ranged, the whole series culminating in considerations surrounding the possibility of a Royal Commission.

### MEDICAL TREATMENT AND POOR-LAW DISQUALIFICATION.

The London County Council has recently been in communication with the Charity Commissioners, as stated in The Hospital on December 19, urging that in certain new schemes which were being drawn up, the disqualification arising from becoming an inmate of a Poor-Law institution for the purpose of receiving medical relief shall not operate until after a residence of four weeks. The Charity Commissioners, however, say that the clause has been adopted after careful consideration, and that they would hesitate to vary it in view of the fact that their experience does not show that it has caused hardship. They express the opinion that if the provision were omitted, the pension, which would continue to be payable, would not go to the recipient, but for the benefit of the institution of which the recipient was an inmate. As the point has cropped up on one or two occasions lately, the Council suggest that it could be met if the schemes were made less mandatory, and a discretion were given to the trustees of the charities affected to continue to pay the pension for a period not exceeding one month after a pensioner has entered a Poor-Law Infirmary for the purpose of receiving medical or surgical treatment.

### THE NEED FOR BEDS AFTER THE WAR.

An argument, not yet insisted on, as to the need for carrying on schemes of extended hospital accommodation on the part of public authorities was put before the Huddersfield Board of Guardians last week by Mr. P. H. Bagenal, the retiring inspector of the Local Government Board. In congratulating the Guardians on their decision to increase the hospital accommodation at the Crosland Moor institution, he said that the war would leave behind it a crop of cases of all kinds requiring institutional treatment. Men would come back with broken constitutions and numbers with rheumatic affections. Mr. Bagenal did not say that there would not be adequate treatment for our soldiers when they came back, but a number of them could not be treated satisfactorily in their own homes, and these would be compelled to go either into the voluntary or the Poor-Law hospitals. The greatest achievement of the Poor-Law reform party, concluded the inspector, had been to concentrate the Guardians' attention on the improvement of hospital accommodation. The nature of this hint should be sufficiently clear, and the prospect foreseen cannot be shirked. There will be an aftermath of cases, verging on the chronic type, which will indeed require treatment in institutions, and it is well to look ahead and recognise the need for it. No doubt to many enthusiasts and eager patriots it seems monstrous to foresee, after all the talk, a Poor-Law institution as the end which may await certain of the eager recruits to-day, but we have already insisted on the fact that the problems of urgency created by the war will do more to bring about that co-operation between the voluntary and the public hospital services which alone can provide adequately for all classes of case and degrees of income. The need is to prepare in advance and abolish the taint of pauperism in any scheme, making use of Poor-Law institutions which may be required.

### HOSPITAL ECONOMY AND ANÆSTHETICS

Now that the "open" method of administering æther is in vogue the consumption of this anæsthetic has increased greatly at many of our hospitals. In this connection it is of interest to note that the British Pharmacopœia, 1914, states that æther may be prepared from either ethylic alcohol or industrial methylated spirit (*i.e.* a mixture of alcohol 19 parts with wood naphtha 1 part) and sulphuric acid. For years it has been the custom at some hospitals to use a purified methylated æther as it has been called, and now that this æther has been accorded "official" sanction in the Pharmacopœia it is likely to be more largely used. From a chemical standpoint, æther of a high degree of purity can be prepared from methylated spirit, and—in the judgment of many anæsthetists who continually use it—its physiological action is all that could be desired. As the difference in cost of the two æthers is about 4s. per pound (due mainly to the Excise duties on ethylic alcohol), it would seem that the question is worthy of the consideration of hospital administrators. We do not, of course, advocate economy without regard to quality, but it may be that here is an opportunity for saving without detriment to the patient. Incidentally, we may note that chloroform, as used at nearly all our large hospitals, is manufactured either from acetone or industrial methylated spirit; it appears to be as satisfa[illegible]ory as that prepared from ethylic alcohol (duty liable). We understand that one large London hospital still uses only chloroform prepared from ethylic alcohol (duty paid), though perhaps this may be due to continuation of a policy adopted before the manufacture and purification of chloroform from industrial methylated spirit had attained the perfection it has now reached. Reminding our readers that we dealt with the price of anæsthetics as a question of economy for hospital boards in The Hospital of November 16, 1912 (p. 181), we should be interested to hear what our hospital anæsthetists think.

### AUSTRALIA'S HOSPITAL UNIT.

A Red Cross boat, carrying Australia's war relief contribution, is probably on the high seas at this moment, writes our Australian correspondent. The "passengers" include ninety-four medical men, 161 army nurses, who rank as officers, and 1,003 others, dispensers, carpenters, skiagraphy and electro-therapy attendants, operating-theatre attendants, orderlies, and cooks with certificates for invalid cookery. On board are five separate hospitals: No. I.—Clearing Hospital (from Tasmania), Lieut.-Col. Dr. W. Giblin in charge; No. II.—First Stationary Hospital (from South Australia), Dr. H. W. Bryant, of Victoria, in charge; No. III.—Second Stationary Hospital (from West Australia), Dr. A. T. White in charge; No. IV.—First General Hospital (from Queensland), Dr. W. Ramsay Smith, of Adelaide, in charge; No. V.—Second General Hospital (from New South Wales), Dr. T. E. Martin in charge, affording in all 1,640 beds. Among the equipment are fourteen motor ambulances, fully furnished, the last of which to be presented was from the retail chemists of Victoria. Another is fitted with *x*-rays, and is really an ambulatory operating-room. The nurses also lead in their profession, at the head of the 160 being Miss Jane Bell, lady superintendent of the Melbourne Hospital.

### THIS WEEK'S DRUG MARKET.

The upward tendency of prices continues to be a feature of the market, and business is good. Cod-liver oil is dearer, and the probability is that prices will advance still further; reports from Norway are not of a very definite character, but it would appear that the fishing up to the present has not been altogether satisfactory; the risks of shipping will also add to the cost of the oil. There is no sign at present of any relaxation of the high prices of salicylates; on the contrary, it seems probable that higher values will be reached before the market is relieved by more plentiful supplies. Emetine is quoted at higher prices in consequence of the advanced value of ipecacuanha. There is practically no change in the position of opium; the strong demand for morphine and codeine is maintained. Camphor has an upward tendency in price, but no serious advance is anticipated. Barbitone, atropine, and cantharidin are dearer. Potassium permanganate is obtainable at rather lower prices, and potassium chlorate is higher. Santonin is likely to be in more plentiful supply, and it might be advisable to delay purchasing for the present.

